# The transition from amalgam to other restorative materials in the U.S. predoctoral pediatric dentistry clinics

**DOI:** 10.1002/cre2.196

**Published:** 2019-06-12

**Authors:** Elham T. Kateeb, John J. Warren

**Affiliations:** ^1^ Department of Periodontics and Preventive Dentistry Al‐Quds University Jerusalem Palestine; ^2^ Department of Preventive and Community Dentistry University of Iowa Iowa City Iowa

**Keywords:** composite resins, dental amalgam, dental schools, pediatric dentistry

## Abstract

Increased concerns about the safety of amalgam restorations in children have resulted in many dental schools emphasizing the teaching of alternative dental materials. This study investigated the current teaching of different dental materials for use in posterior teeth in the United States predoctoral pediatric dentistry programs. In 2011, the authors invited the chairs of the predoctoral pediatric dentistry departments in all accredited dental schools at that time (*N* = 57) to participate in an internet‐based survey. Descriptive statistics were calculated to describe the frequency of using different restorative materials. Regression models were developed to explore the factors related to the use of dental restorations in predoctoral pediatric clinics. Among the 44 dental schools that responded (77% response rate), 74% used amalgam, and 93% used composite in primary posterior teeth. Glass ionomer was used by 61% of the schools in primary posterior teeth. Placing amalgam in primary posterior teeth was associated with programs that treated more 3–5‐year‐old patients (β = .302, *p* < .043), whereas the use of glass ionomer was associated with having students serving at off‐site satellite dental clinics (β = .015, *p* < .012). In general, having departments with chairs who had positive attitudes towards Minimal Invasive Dentistry (MID) used composite (β = .091, *p* < .0001) and glass ionomer (β = 103, *p* < .0001) more frequently and were less likely to use amalgam (β = −.077, *p* < .005) in primary posterior teeth. Although teaching MID concepts in predoctoral pediatric clinics in dental schools is increasing, the use of amalgam in posterior primary and permanent teeth is still widely practiced.

## 1 INTRODUCTION

Although much progress has been made in its prevention, dental caries is still one of the most common chronic diseases worldwide (FDI World Dental Federation, 2014). Dental caries, when not treated in its early stages, usually progresses to dental cavities that need to be restored with dental filling materials. Dental filling materials vary in their durability, compatibility with human tissues, and safety. Dental amalgam, which contains 50% mercury, is a restoration material that has been in use to restore dental cavities for over 150 years and has the longest life expectancy among all direct restorative materials. Its ease of use, appropriate mechanical and bacteriostatic properties, and cost‐effectiveness have made dental amalgam the material of choice to restore dental cavities especially among high‐risk populations (FDI World Dental Federation, 2014). Many advances in developing new dental materials with better esthetic characteristics have occurred; however, no universal substitute is currently available (FDI World Dental Federation, 2014).

The Minamata Convention on Mercury, a global treaty governing the mining use and trade in mercury, has agreed on 2013 to a worldwide reduction and ultimate elimination in the production and use of mercury containing products (Minamata Convention on Mercury, n.d.). The Convention called for a phase‐down approach to dental amalgam through greater emphasis notably on prevention, research into new dental materials, and best management practices (WHO consensus statement on dental amalgam, 1997).

To comply with Minamata convention, a consensus statement on dental amalgam by the International Dental Federation, FDI, and the World Health Organization (WHO) called for phasing down the use of amalgam fillings mainly due to environmental concerns (FDI World Dental Federation, 2014; WHO consensus statement on dental amalgam, 1997). The statement, at the same time, affirmed that “the current weight of evidence indicates that contemporary dental‐restorative materials, including dental amalgam, are considered to be safe and effective” (WHO consensus statement on dental amalgam, 1997).

The American Dental Association confirmed that dental amalgam has been studied and reviewed extensively and has established a record of safety and effectiveness (American dental association, n.d.). However, the United States has signed and offered acceptance of the Minamata Convention documents in November 2013, joining other nations in moving the legally binding treaty forward (ADA News, 2013).

One of the measures that was suggested by Minamata Convention to phase down the use of mercury is to “encourage dental schools to educate and train dental professionals and students on the use of mercury‐free dental restoration alternatives and promoting best management practices” (Minamata Convention on Mercury, n.d.).

Dental schools worldwide adopted different strategies to change their teaching philosophies from traditional amalgam restorations to more minimally invasive techniques such as using composite‐based restorations and glass ionomers. Previous literature (Mjør & Wilson, 1998; Wilson, 1989; Wilson & Mjør, 2000) from the end of the last century demonstrated that, in general, dental schools in North America and Europe have tended to increase the teaching of composite‐based restorations in restoring posterior teeth. These data showed considerable variations within and between countries. Some countries having discontinued the teaching of the use of dental amalgam in most, if not all of its dental schools, whereas in other countries, there has been relatively little movement away from amalgam and, as a consequence, limited curriculum time devoted to the use of composite restorations to restore posterior teeth.

A more recent study in a U.S. dental school found that the number of preclinical lecture and simulation laboratory sessions spent on teaching amalgam restorations' preparation and placement were 2.5 times greater than the number of sessions devoted to teach composite restorations (Ottenga & Mjør, 2007). However, in clinic, composite restorations were used to restore posterior teeth at a rate that was 2.3 times more often than that of amalgam. The only instance that students were instructed to use amalgam over composite in clinics was in restoring four‐surface posterior cavities.

In another study that was conducted in 2009, the authors investigated the current teaching of posterior composite in 67 U.S. and Canadian dental schools. Forty‐nine schools completed the online survey and demonstrated that although all schools taught the placement of resin‐based composites in occlusal and most occlusoproximal cavities, eight schools (16%) did not teach placement of three‐surface occlusoproximal resin‐based composite restorations in permanent molars (Lynch, Frazier, McConnell, Blum, & Wilson, 2011). The same study showed that resin‐based composites accounted for 49% of direct posterior restorations placed by dental students in the academic years of 2009 and 2010, a 30% increase from 2005.

A more recent study in 2015 compared teaching time with students' clinical procedures in amalgam and composite posterior restorations in dental schools across the United States. Of the 60 dental schools, 12 returned surveys with complete data. Findings from this preliminary study reflected a small increase in two‐surface resin‐based restorations placed by dental students from 2009 to 2011 and little change in curricular time devoted to teaching amalgam restorations. However, the total number of posterior composite restorations placed by students in these schools was slightly higher than amalgams (Rey, Nimmo, Childs, & Behar‐Horenstein, 2015).

Most of the previous studies assessed the teaching of composite and amalgam restorations in Operative Dentistry lab and clinics. One study in Canada compared the teaching of amalgam and composite‐based restorations in posterior teeth between Operative Dentistry and Pediatric Dentistry undergraduate clinics (McComb, 2005). A 10‐question survey was mailed to 10 Canadian faculties of Dentistry. The results from 10 pediatric dentistry and eight restorative programs showed that the relative emphasis on the two materials varied. In the operative programs, curriculum time devoted to silver amalgam was either greater than or equal to that devoted to posterior composite. Whereas five of the eight schools reported greater educational emphasis on silver amalgam for the permanent dentition. The responses from the pediatric dentistry programs were more diverse. Five schools reported more emphasis on silver amalgam, three schools reported equal emphasis, and two schools reported more emphasis on posterior composite (McComb, 2005).

Although a reasonable amount of data is available to describe the trends in teaching amalgam and composite in undergraduate students clinics, very little is known about the teaching philosophies used to restore posterior teeth in predoctoral pediatric dentistry programs in U.S. dental schools. Thus, the purpose of this study was to investigate the current teaching of different dental materials in posterior teeth in predoctoral pediatric dentistry programs in the United States, especially amalgam and composite‐based restorations.

## 2 METHODOLOGY

The current analysis is a part of a larger study that assessed predoctoral pediatric dentistry programs' use of different MID techniques. The survey was pretested for content validity, using cognitive analysis (consulting and pretesting the instrument with experts) by six faculty members from the Department of Preventive and Community Dentistry, four faculty members from the Department of Pediatric Dentistry, and one faculty member from the Department of Operative Dentistry, all at the University of Iowa. Pilot testing for face validity was carried out by two pediatric dentistry senior residents and two dental public health senior residents, also from the University of Iowa. Submitting a completed questionnaire constituted the subjects' consent. The study was approved by the University of Iowa Institutional Review Board.

A list of pediatric dentistry department chairs in the U.S. dental schools was obtained from the American Academy of Pediatric Dentistry and was verified by the American Dental Association's list of accredited dental schools as of April 2010. The survey was administered using an online software and was sent to the program chairs in 57 dental schools in the end of 2011. Two follow‐up surveys were e‐mailed to nonrespondents 2 and 4 weeks after the first e‐mail.

In addition to asking program chairs about the use of different restoration materials and techniques to manage dental caries among pediatric patients, characteristics and demographics of their programs and the patient population they serve were investigated.

The dependent variables in this analysis were the use of different restoration materials—amalgam, composite‐based materials and glass ionomers—were measured on a 5‐point scale (never = 1 to very often = 5).

In order to get a more parsimonious design and minimize the number of variables that would be used in the final regression model, an attitude towards MID scale (composite variables) was constructed from this survey. The program directors' attitude towards MID was used as a predictor variable to explain the use of different restoration materials. The agreement or disagreement of program directors with statements about MID concepts was measured on a 5‐point Likert scale for seven subquestions. The scale summed the scores for each subquestion, ranging from 1 = *strongly disagree* to 5 = *strongly agree*. Therefore, the most negative attitude would be scored as 7, and the most positive attitude would be scored 35 on this scale. The scale had a Cronbach's alpha of .76, and the mean for the study sample was 29 ± 4. The “Attitude Towards MID” scale consisted from statements about, (1) using the fluoride as a re‐mineralizing agent, (2) carrying out periodical risk assessment, (3) placing fissure sealants at insipient carious lesion, (4) excavating caries with hand excavator, (5) using Atraumatic Restorative Treatment (ART) as a valid practice, (6) definitive restorations are not always the treatment of choice, (7) leaving being always caries in the floor of a prepared cavity sometimes is justified. Other key independent variables included in the analyses are shown in Tables 1 and 2.

**Table 1:** Respondents predoctoral pediatric dentistry programs characteristics

Predoctoral pediatric dentistry program characteristics (43/44 institutions answered this question)	Frequency	Valid (%)
Dental school main location		
Urban area—inner city	28	65
Urban area but not located in the inner city	7	16
Urban area, suburb	2	5
Small city	6	14
Rural or small town	—	—

Percentage of dental students' time spent in each of the following settings		
	Mean	SD
On‐site dental school facilities	71.5	27
Affiliated hospital‐based dental clinics	5	9
Off‐site, satellite, or affiliated dental clinics	14	22
Off‐site, public health clinics	9	19
Off‐site, migrant worker camps	0.05	0.3
Off‐site, international programs	0.4	2

**Table 2:** Characteristics of patient population served by the predoctoral pediatric dentistry program

Patients population's characteristics	Mean (%)	SD (%)
Covered by Medicaid and other public insurance	64	26
Covered by private insurance	12	12
Have no insurance (out of pocket)	20	22
Proportion of high‐risk children treated in pediatric dentistry residency programs	63	20
Proportion of low risk children	27	13
Proportion of children younger than 3 years treated	6	6
Proportion of 3–5 years children treated	20	18
Proportion of 6–12 years children treated	57	18
Proportion of children 13 and older treated	20	16

The data were exported into SPSS data files, and the IBM SPSS 20 (IBM Corp, 2011) was used to carry out the analysis. Statistical analyses included descriptive statistics to describe sample characteristics, bivariate analyses to explore associations between predictor and outcome variables, and multivariable modeling to assess the variables that may explain our three outcome variables. Three separate models were built for the dependent variables, “Placing amalgam in posterior primary teeth,” “Placing composite‐based restorations in posterior primary teeth,” and “Placing Glass Ionomer in posterior primary teeth”. Stepwise and backward multiple linear regression were used to assess relationships of predictor variables with our dependent variables.

## 3 RESULTS

A response rate of 77% was obtained (44 predoctoral pediatric dentistry programs). Response bias was assessed by comparing respondent and nonrespondent programs in a descriptive way according to variables obtained from American Dental Education Association (ADEA) dental school profile; no response bias was detected.

Twenty‐eight dental schools out of the 44 were located in inner cities, and 29% of students' time in pediatric dentistry clinics was devoted to off‐campus locations such as satellite clinics and migrant camps. Participating dental schools characteristics are found in Table 1.

Sixty‐three percent of the children's population served by dental schools in our sample were high caries risk, 64% were covered by Medicaid, and 20% had no insurance. Seventeen dental schools (39.5%) from our sample routinely treated children with special health care needs. Patients' population served by the 44 dental schools characteristics are found in Table 2.

**Figure 1:**
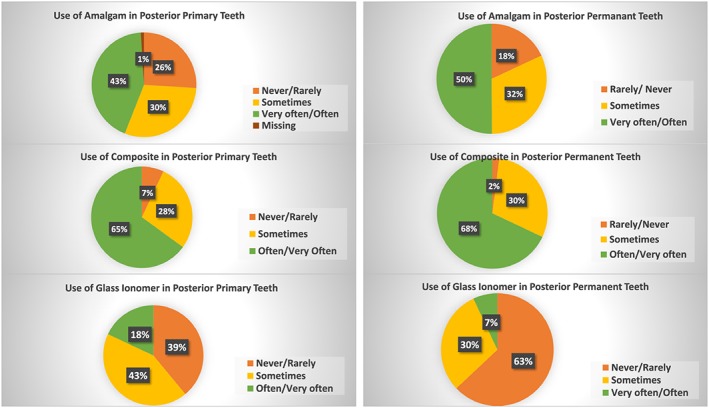
The use of different dental materials in predoctoral pediatric dentistry clinics

**Figure 2:**
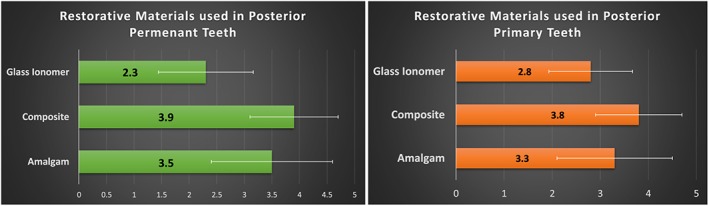
Mean rating of the use of amalgam, composite, and glass ionomer in predoctoral pediatric dentistry clinics

Thirty‐two dental schools (74%) used amalgam in primary posterior teeth, and 36 dental schools (82%) used it in permanent teeth. In contrast, 40 dental schools (93%) used composite in posterior primary teeth, and 43 schools (99%) used it in posterior permanent teeth. Glass ionomer was used by 27 dental schools (61%) in primary posterior teeth and by 16 schools (37%) in permeant posterior teeth. The use of different restorative materials in predoctoral pediatric dentistry clinics is shown in Figures 1 and 2.

Twelve dental schools (27%) used G.V. Black “Extension for Prevention” concept in teeth preparation (often/very often), and 21 dental schools (47.7%) used this concept (rarely/never). On the other hand, one out of the 42 dental schools (2.3%) used a very conservative restorative technique, the Hall technique, which consisted of “crowning vital, decayed, asymptomatic primary molars without preparation.”

Out of 16 dental schools, three programs (18.8%) sent children 3 years and younger to the operating rooms (often/very often) to receive dental treatment.

Placing amalgam in primary posterior teeth was associated with programs that used fewer MID techniques in their clinical training, such as composite and glass ionomer in posterior teeth (ρ = −.36, *p* = .02 and ρ = −0.33, *p* = .028) and used G.V. Black “Extension for Prevention” philosophy more often (ρ = .46, *p* = .002). Also programs that treated fewer children without insurance used amalgam in posterior primary teeth more often (ρ = −.36, *p* = .02). However, placing amalgam in permanent posterior teeth was associated with programs that considered “Child's caries risk” as an unimportant factor in selecting dental restorative materials (ρ = −.35, p = .02), programs that treated fewer children without insurance, (ρ = −.33, p = .02), used “Extension for Prevention” philosophy (ρ = .56, *p* < .0001) more often and used composite‐based restorations in posterior teeth less often (ρ = −.45, *p* = .002). On the other hand, placing composite in primary and permanent posterior teeth was associated with programs that considered “Child's caries risk” as a very important factor in selecting dental restorative materials (ρ = .32, *p* = .04 and ρ = .33, *p* = .03).

The use of glass ionomer in primary posterior teeth was associated with programs that spent less time on clinical training in “on‐site” dental school facilities (ρ = −.36, p = .03). Whereas, the use of glass ionomer in permanent posterior teeth was associated with programs that consider “Child's caries risk” as an important factor in selecting dental restorative materials (ρ = .356, *p* = .02).

In the final models, placing amalgam in primary posterior teeth was associated with programs that treated more 3–5‐years‐old patients (β = .302, *p* < .043), whereas the use of glass ionomer in primary posterior teeth was associated with having students serving at off‐site satellite dental clinics (β = .015, *p* < .012). In general, programs that had directors with positive attitudes towards MID used Composite (β = .091, *p* < .0001) and glass ionomer (β = .103, p < .0001) more and used amalgam (β = −.077, *p* < .005) less in primary posterior teeth.

## 4 DISCUSSION

In response to the calls for phasing down in the use of amalgam, dental schools in the United States face the ongoing challenge of having an evidence‐based and up‐to‐date curriculum with respect to the developments and changes in dental practice around the world.

Reports from dental schools in the United States, Canada, Ireland, and the United Kingdom over the past two decades (Lynch, McConnell, & Wilson, 2006; WHO consensus statement on dental amalgam, 1997) have found an increase in the teaching of dental materials other than amalgam, such as posterior composites. However, in Europe, the shift has been more evident than in the United States, which seems to be lagging behind in embracing this global trend. The assessment of what is taught in dental schools is a very important first step in any plan to advocate for curricula changes.

This study is important for three reasons: first, it covered a point of time where no other reports documented the degree of amalgam instruction in U.S. dental schools (circa 2011). Second, it assessed the use of amalgam in pediatric patients, a population in which a total ban of amalgam use has been called for in Europe (Dental Tribune International, 2016). Third, 44 pediatric dentistry departments completed the survey—a response rate (77%) higher than in all other published reports.

Results of the current study showed that, although the use of composite restorations in predoctoral pediatric clinics was greater than amalgam in both primary and permanent posterior teeth, amalgam use was not far behind. This indicates that the transition is still in its early stages. Additionally, it was interesting that the “Extension for Prevention” technique was still used widely in posterior primary teeth compared with composite and glass ionomer or more conservative techniques. Although it was a revolution in its time given the dental materials available, the new concepts of cavity preparation now that are based on the advanced diagnostic equipment, new restorative materials, and our current understanding of the biology of caries, makes “extension for Prevention” very outdated (Hamama, Yiu, & Burrow, n.d.).

Amalgam was used widely in the current study. One explanation of the high use of amalgam in the current study can be the uniqueness of the patient population served by dental schools in general and in our sample in particular. Usually, high caries risk patients with no insurance or Medicaid patients constitute the majority of this population. This is in line with our study results; schools who treated more children without insurance used more amalgam restorations in their pediatric clinics. The low cost of amalgam was a big plus that made it popular in high‐risk patients who lack insurance to cover their treatment costs. However, this may change in response to the environmental fees associated with the use of amalgam in private practices resulting from the Clean Water Acts (Rey et al., 2015).

Our results showed that predoctoral pediatric dentistry departments whose leaders had less positive attitudes towards MID concepts, and did not practice MID procedures in teaching their students (often/very often), used more amalgam restorations in their clinics. This attitude towards amalgam is understandable given the long history of this material, and although the science and physical properties of adhesive materials, especially composite, has been progressing rapidly, amalgam still has some advantages over posterior composite with regard to physical properties. In a report by Overton and Sullivan, data showed that posterior composite restorations placed by dental students were replaced 10 times more frequently than amalgam restorations (Overton & Sullivan, 2012). Marginal integrity, durability, wear resistance, and forgiveness in unideal moisture control situations in amalgam restorations are still unmatched by posterior composite (Mjør & Wilson, 1998; Overton & Sullivan, 2012; Wilson, 1989).

Data for this study were collected in the end of 2011 as part of a larger study that assessed different restoration materials and techniques among predoctoral pediatric dentistry clinics. In the few years following this survey, eight new dental schools were opened in the United States. Most of these schools adopted a different model of clinical training than the original model assessed in this study. In our study, 70% ± 26% of the training time was held in the campus' dental facilities, whereas the newer schools have trained more students in health community centers. This shift in the philosophy of training dental students and engaging them more in high risk populations may influence the selection of dental materials. It would be interesting to study the influence of this shift on the use of different dental restoration materials.

It is vital for dental schools to routinely evaluate their curricula to incorporate advances in dental sciences and to respond to developments throughout the world to best prepare new dentists and improve oral health. The findings of this study suggest that the selection of different dental materials among pediatric dental patients is still widely varied, and amalgam is commonly used. Moreover, dental students need to learn the risk assessment approach in selecting the best dental material in restoring children's teeth. Encouragingly, 35 dental schools (85.4%) in our sample used risk assessment with every new pediatric patient. This approach teaches the students to incorporate the best evidence available, their clinical judgment, and the patients' needs in the final treatment decisions.

In summary, the current MID techniques offer great options for tooth structure preservation, such as repair rather than replacement of composite (Gordan et al., 2011), the atraumatic experience associated with glass ionomer restorations in Atraumatic Restorative Technique (ART) (Banerjee, 2018), and the use of Silver Diamine Fluoride as a nonrestorative option to asymptomatic cavities (Slayton et al., 2018). However, U.S. dental schools in the time of the current study did not appear ready to phase out training their dental students on the use of amalgam. It is crucial to repeat this survey in the near future to assess any changes in the curriculum about amalgam teaching. However, until then, it seems that amalgam is still taught widely in predoctoral pediatric dentistry programs as a restoration in primary and permanent posterior teeth. At a policy level in the United States, the American Dental Association's position paper on dental amalgams did not discourage the use of amalgam restorations and confirmed its safety and validity as a current restoration material option (American Dental Association, Council on Scientific Affairs, 2009).

## CONFLICT OF INTEREST

The authors declare no conflict of interest.

## DISCLAIMER

Nothing to declare.

## FUNDING INFORMATION

This project was funded by NIH/NIDC R T32 Grant DEO 14678‐06.
